# Interplay among malnutrition, chemoprevention, and the risk of malaria in young Ugandan children: Longitudinal pharmacodynamic and growth analysis

**DOI:** 10.1002/psp4.12892

**Published:** 2023-03-14

**Authors:** Ali Mohamed Ali, Erika Wallender, Emma Hughes, Grant Dorsey, Radojka M. Savic

**Affiliations:** ^1^ Department of Bioengineering and Therapeutic Sciences University of California San Francisco San Francisco California USA; ^2^ Bagamoyo Research and Training Center Ifakara Health Institute Bagamoyo Tanzania; ^3^ Department of Clinical Pharmacy University of California, San Francisco San Francisco California USA; ^4^ Department of Medicine University of California, San Francisco San Francisco California USA

## Abstract

African children are at risk of malaria and malnutrition. We quantified relationships between malaria and malnutrition among young Ugandan children in a high malaria transmission region. Data were used from a randomized controlled trial where Ugandan HIV‐unexposed (*n* = 393) and HIV‐exposed (*n* = 186) children were randomized to receive no malaria chemoprevention, monthly sulfadoxine‐pyrimethamine, daily trimethoprim‐sulfamethoxazole, or monthly dihydroartemisinin‐piperaquine (DP) from age 6–24 months, and then were followed off chemoprevention until age 36 months. Monthly height and weight, and time of incident malaria episodes were obtained; 89 children who received DP contributed piperaquine (PQ) concentrations. Malaria hazard was modeled using parametric survival analysis adjusted for repeated events, and height and weight were modeled using a Brody growth model. Among 579 children, stunting (height‐for‐age z‐score [ZHA] < −2) was associated with a 17% increased malaria hazard (95% confidence interval [CI] 10–23%) compared with children with a ZHA of zero. DP was associated with a 35% lower malaria hazard (hazard ratio [HR] [95% CI], 0.65 [0.41–0.97]), compared to no chemoprevention. After accounting for PQ levels, stunted children who received DP had 2.1 times the hazard of malaria (HR [95% CI] 2.1 [1.6–3.0]) compared with children with a ZHA of zero who received DP. Each additional malaria episode was associated with a 0.4% reduced growth rate for height. Better dosing regimens are needed to optimize malaria prevention in malnourished populations, but, importantly, malaria chemoprevention may reduce the burden of malnutrition in early childhood.

**Study Highlights**

**WHAT IS THE CURRENT KNOWLEDGE ON THE TOPIC?**

The greatest burdens of malnutrition and malaria overlap in sub‐Saharan Africa, but little is known about the relationships among malnutrition, malaria, and the efficacy of malaria chemoprevention.

**WHAT QUESTION DID THIS STUDY ADDRESS?**

We used parametric survival and growth models to answer whether malnutrition is associated with an increased hazard of malaria and whether malaria is associated with decreased childhood growth.

**WHAT DOES THIS STUDY ADD TO OUR KNOWLEDGE?**

We show that malnutrition severity is associated with an increased hazard of incident malaria even when effective malaria chemoprevention is administered. Additionally, our findings indicate that each additional episode of malaria is associated with a reduced growth rate for height.

**HOW MIGHT THIS CHANGE DRUG DISCOVERY, DEVELOPMENT, AND/OR THERAPEUTICS?**

This study highlights the importance of including malnourished children in clinical trials. Stunted children have a higher risk of malaria regardless of chemoprevention regimen, suggesting that more work is needed to optimize chemoprevention regimens for this population. Future clinical trials should (1) be powered to evaluate malnutrition as a covariate and (2) incorporate evaluation of nonmalarial outcomes, including malnutrition/childhood growth.

## INTRODUCTION

Malaria and malnutrition are significant causes of morbidity and mortality in young children in sub‐Saharan Africa. Children less than 5 years of age accounted for 77% of malaria deaths globally in 2020,^1^ and malnutrition is present in nearly half of all young children who die.^2^, ^3^ The estimated 58.7 million malnourished children residing in Africa are frequently also at high risk of falciparum malaria.^4^ Although the burden of malaria and malnutrition overlap, a relationship between these conditions has been difficult to quantify as large cohorts with paired longitudinal malaria incidence and frequently measured anthropometric data are rarely available.

Chronic malnutrition, as measured by low height‐for‐age z‐score (ZHA), in young African children has been associated with increased risk of malaria, severe malaria, and anemia.^5^, ^6^, ^7^, ^8^ Proposed mechanisms for this observation include lower antimalarial immunity,^9^ lower exposure to antimalarial drugs due to poor absorption or poorly assigned weight‐bands for pediatric dosing,^10^, ^11^ or less access to malaria control measures.^12^, ^13^, ^14^ Despite these observations, it is unclear how best to incorporate malnutrition in malaria chemoprevention or other malaria control efforts. Quantifying the contribution of malnutrition to malaria risk is key to reducing the malaria burden in young African children.

It is also likely that malaria exacerbates malnutrition. Use of long‐lasting insecticide treated bed nets (LLINs) for malaria prevention led to greater weight and height gains in children when compared to no LLIN use.^15^ However, the majority of studies that examined relationships between malaria and risk of malnutrition showed no relationship. These studies had important limitations, including infrequent anthropometric measurements, lack of malaria incidence data, or cohort enrollment in regions with seasonal or low malaria transmission.^16^, ^17^ Malaria likely worsens childhood malnutrition to the greatest degree in regions with high perennial transmission. Resolving uncertainty around the interplay between malaria and malnutrition would allow us to better target malaria control and nutritional interventions.

We identified and quantified risk factors for malaria and malnutrition in a large cohort of Ugandan children who were followed from 6 months to 3 years of age as part of two randomized controlled trials of three malaria chemoprevention regimens.^18^, ^19^ Using this unique dataset with frequent anthropometric measurements, comprehensive covariate data, including drug concentrations, and accurate malaria incidence measurement, we used quantitative modeling techniques to estimate how changes in malaria burden or malnutrition impact future growth and malaria incidence, respectively. We conducted simulations to quantify the relationship between malaria and childhood growth.

## METHODS

### Study design and participants

Data from two randomized controlled malaria chemoprevention trials conducted concurrently in the same clinic in Tororo, Uganda, from June 2010 through September 2013 were used.^18^, ^19^ Eligible children were 4–5 months of age, lived within 30‐km of the study clinic, and were HIV‐uninfected with known HIV status of the mother. One trial enrolled HIV‐unexposed children (the mother was HIV‐uninfected) and one trial enrolled HIV‐exposed children (the mother was HIV‐infected). At enrollment every household was given two LLINs and the caregiver participated in a sociodemographic survey. These studies were approved by ethics committees at Makerere University, the Ugandan National Council of Science and Technology, and the University of California, San Francisco, and a signed written informed consent form was obtained from all parents/guardians. Both studies were listed under the same clinical trial registration number: NCT00948896.

### Study procedures

HIV‐unexposed children began chemoprevention at 6 months of age and, because HIV‐exposed participants received daily trimethoprim‐sulfamethoxazole (TS) while breastfeeding per national guidelines, they began chemoprevention at 6 months of age or upon breastfeeding cessation, whichever was later. All participants received chemoprevention until 24 months of age and were followed off chemoprevention until 36 months of age. Children were randomized in equal numbers to no chemoprevention, a once monthly dose of sulfadoxine‐pyrimethamine (SP), a daily dose of TS, or a monthly treatment course (daily dosing for 3 consecutive days) of dihydroartemisinin‐piperaquine (DP). Each regimen was administered according to weight‐bands specified by the manufacturer at the time of the study (Table S4 and Figure S6).^18^, ^19^ Parents and guardians were given supplies of the study drugs to be taken unobserved at home and accurate adherence data were not available.

Children received all medical care at a dedicated study clinic to ensure accurate diagnosis of incident malaria. Children returned monthly for weight and height measurements and the parent/guardian was asked if the child had breast fed in the last 7 days. At any visit, if a child had a fever (>37.5°C) or a history of fever within the last 24 h, a thick blood smear was taken to evaluate for malaria. If the blood smear was positive, the child was diagnosed with and treated for malaria. The time of incident malaria was defined as the day of a visit where malaria was diagnosed which occurred greater than 14 days after a prior episode.

Monthly age, weight, and height measurements were used to calculate weight‐for‐age z‐score (WAZ), ZHA, and weight‐for‐height z‐score (WHZ). Z‐scores were calculated using the World Health Organization's (WHO) Anthro macro.^20^, ^21^ A child was considered underweight, stunted, or wasted if they had a WAZ less than −2, ZHA less than −2, or WHZ less than −2, respectively. Z‐scores considered physiologically implausible by the WHO macro were excluded from the analysis (Supplemental Methods).

### Piperaquine assay and piperaquine concentration data

A subset of HIV‐unexposed children who received DP underwent sampling of venous plasma to determine piperaquine (PQ) concentrations.^22^ PQ concentrations were measured either at malaria diagnosis or at routine study visits at 4 month intervals using a validated liquid chromatography and tandem mass spectrometry method.^23^ As DP was taken at home without directly observed therapy, PQ concentrations were not linked to specific dosing events in this analysis. The plasma assay had a quantification range of 1.5–250 ng/ml with 1.5 ng/ml as the lower limit of quantification (LLOQ).

### Parametric survival analysis

A parametric survival model, adjusted for repeated events, was developed to estimate the hazard of incident malaria from 6 to 24 months of age, during chemoprevention. The Laplace estimation method in NONMEM, version 7.4.2 was used.^24^ The baseline hazard was estimated for all children, and individually by treatment arm. Exponential, Weibull, and Gompertz distributions were tested as the baseline hazard model. Events were right censored at the participant's last visit if before 24 months of age, or at the end of chemoprevention at 24 months. Covariates were tested on baseline hazard using stepwise covariate modeling (SCM) with a *p* value of less than 0.05 for forward inclusion and a *p* value of less than 0.01 for backward elimination using linear and exponential functions.^24^ Covariates determined at the start of chemoprevention included sex, chemoprevention regimen, HIV exposure status, use of other malaria prevention methods (keeping doors and windows closed in the evening, emptying/covering water sources assessed at the start of the study), and derived socioeconomic tertile. Time‐varying covariates included age (as HIV‐exposed and ‐unexposed children could begin chemoprevention at different ages), weight, WAZ, ZHA, and WHZ. If malnutrition status was missing at a routine visit, the prior measurement was carried forward. HIV exposure status, chemoprevention arm, use of malaria prevention measures, and socioeconomic tertile were available for all individuals.

Model selection was guided by change in objective function value (OFV) between nested models, parameter estimates, relative standard error (RSE), and Kaplan–Meier visual predictive check.^25^, ^26^

Hazard ratios (HRs) with 95% confidence intervals (CIs) were calculated using the final parameter estimates and covariance matrices. Percent reductions in incident malaria hazard were defined as 1 – the HR. For the final models, 95% CIs of parameter estimates were determined by bootstrap (*n* = 1000).

### Sub‐analysis using PQ concentration data

Drug concentration data were available for a subset of 89 HIV‐unexposed children, and a stand‐alone parametric survival model was developed to assess the impact of PQ concentration on malaria hazard among the subset of children with at least one PQ concentration. After baseline model selection, covariate analysis using SCM was performed as described above. All covariates tested are listed in the Supplementary Methods.

### Growth models

Growth models were developed to predict height and weight from 6 months to 3 years of age. Monthly height, weight, and age measurements were described using a Brody growth model (Equations listed in Supplementary Methods). Covariate relationships were also explored for the baseline height/weight, the maximum height/weight, and growth rate (KTR).

(1) $$\mathrm{KTR}=\alpha \times {\mathrm{Age}}^{\beta }\times e^{\eta }$$

The equation for growth rate is shown in Equation 1, where α is the typical growth rate, β is the parameter for the age function, and *η* is the residual error. Covariates included those listed above as well as time varying cumulative number of malaria episodes, and whether or not the child was breast fed in the last 7 days. If an individual was lost to follow‐up, their data were included until they left the study. If height or other covariate data was missing at a routine visit, the prior measurement was carried forward. For the final model, 95% CIs of the parameter estimates were determined by bootstrap (*n* = 1000).

### Simulations

The final survival models were used for simulations (*n* = 1000) to predict the total number of malaria episodes in stunted (ZHA = −2.1) and non‐stunted (ZHA = 0) children. For simulations, children were assumed to be HIV‐unexposed and in the low or middle socioeconomic category. For the DP arm, we conducted 1000 simulations of the above patients, but stratified into groups with PQ concentrations consistently greater than 10.3 ng/ml or less than or equal to 10.3 ng/ml.^27^ A sensitivity analysis with higher PQ target concentrations is described in the Supplementary Results.^28^, ^29^

Simulations using the final growth model for height were used to predict the proportion of stunted (ZHA = −2.1) children at 3 years of age, stratified by cumulative malaria episodes. Four datasets comprised of baseline WHZ, ZHA, sex, HIV exposure status, and time varying cumulative malaria episodes (0, 5 [lower 25th percentile of data], 11 [median of data] or 17 [upper 75th percentile of data]) from 6 months to 3 years of age were simulated 100 times. Predicted ZHA was calculated using the WHO Anthro macro.^21^

## RESULTS

### Study participants and raw data

**FIGURE 1:**
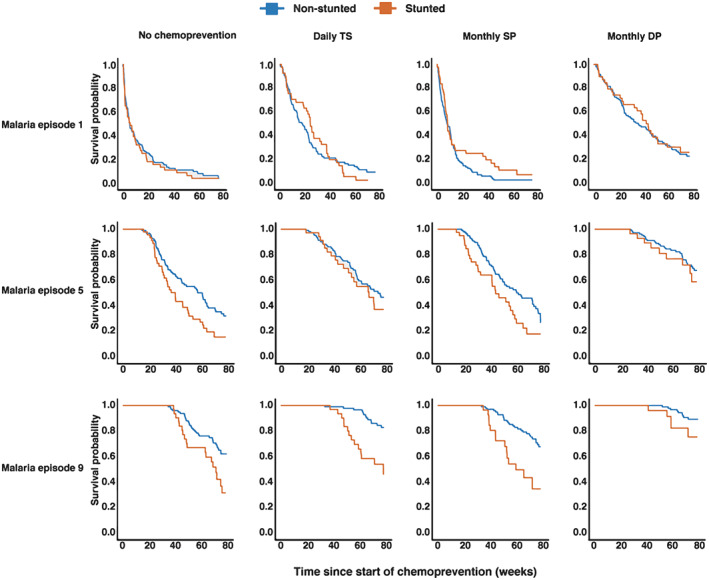
Kaplan‐Meier curves stratified by chemoprevention and nutritional status (stunted: height‐for‐age z‐score < −2 [orange line]; non‐stunted: height‐for‐age Z‐score ≥ −2 [blue line]). Time indicates time from beginning of chemoprevention to the subsequent malaria episode. DP, dihydroartemisinin‐piperaquine; SP, sulfadoxine‐pyrimethamine; TS, trimethoprim‐sulfamethoxazole.

Among the 579 children included in the analysis, 186 (32.1%) were HIV‐exposed. HIV‐exposed children were older when they began study drugs compared with HIV‐unexposed children (median age: 10 months vs. 6 months) and had lower median Z‐scores (Table S1). In total, 526 children were followed to the end of chemoprevention through 24 months of age (352 HIV‐unexposed and 174 HIV‐exposed), and 511 children were followed to 36 months of age (340 HIV‐unexposed and 171 HIV‐exposed). Baseline characteristics stratified by chemoprevention regimen did not differ (Table 1). Malaria outcomes by chemoprevention arm have been previously reported.^18^, ^19^ Briefly, 493 (85.1%) children experienced at least one malaria episode. During chemoprevention from 6–24 months of age, children in the no chemoprevention and SP arms had the highest number of malaria episodes (Table S2). After the first malaria episode, stunted children (ZHA < −2) had an increased hazard of subsequent incident malaria for all treatment arms (Figure 1) and regardless of HIV exposure status (Figure S1).

**TABLE 1:** Characteristics of study participants at the start of chemoprevention

Characteristic^a^	No chemoprevention	Daily TS	Monthly SP	Monthly DP
*N*	144	146	144	145
Female, *n* (%)	70 (48.6)	75 (51.4)	65 (45.1)	77 (53.1)
HIV‐exposed,^b^ *n* (%)	46 (31.9)	47 (32.2)	46 (31.9)	47 (32.4)
Age in months				
HIV‐unexposed	6.1 (6.0, 6.2)	6.1 (6.0, 6.1)	6.1 (6.0, 6.4)	6.1 (6.0, 6.3)
HIV‐exposed	10.0 (7.6, 16.1)	11.6 (7.4, 18.0)	9.3 (6.8, 18.4)	10.3 (6.5 17.6)
Weight in kg	7.2 (5.7, 10.0)	6.7 (5.3, 9.8)	7 (5.3, 9.7)	6.8 (5.7, 9.6)
Weight‐for‐age z‐score	−0.39 (−2.8, 1.4)	−0.58 (−3.2, 1.6)	−0.53 (−3.3, 1.3)	−0.51 (−3.2, 1.3)
Height‐for‐age z‐score	−1.26 (−3.6, 1.0)	−0.80 (−4.0, 1.5)	−1.26 (−3.9, 0.9)	−1.23 (−4.2, 0.9)
Weight‐for‐height z‐score	0.42 (−1.7, 2.4)	−0.02 (−2.6, 1.9)	0.25 (−1.9, 2.1)	0.18 (−2.1, 2.3)
Underweight,^c^ *n* (%)	14 (9.7)	20 (13.7)	21 (14.6)	18 (12.4)
Stunted,^c^ *n* (%)	37 (25.7)	33 (22.6)	40 (27.8)	34 (23.5)
Wasted,^c^ *n* (%)	2 (1.4)	8 (5.5)	3 (2.1)	5 (3.5)
Income strata, *n* (%)				
Low	44 (30.5)	53 (36.3)	52 (36.1)	66 (45.5)
Middle	59 (41.0)	44 (30.1)	49 (34.0)	42 (29.0)
High	41 (28.5)	49 (33.6)	43 (29.9)	37 (25.5)
Other malaria prevention,^c^ *n* (%)	32 (22.2)	33 (22.6)	35 (24.3)	46 (31.7)

Abbreviations: DP, dihydroartemisinin‐piperaquine; SP, sulfadoxine‐pyrimethamine; TS, trimethoprim‐sulfamethoxazole.

^a^ Continuous variables shown as median, (2.5th and 97.5th percentile).

^b^ HIV‐exposed indicates children who are HIV‐uninfected but were born to HIV‐infected mothers.

^c^ Underweight: weight‐for‐age z‐score < −2; Stunted: height‐for‐age z‐score < −2; Wasted: weight‐for‐height z‐score < −2; Other malaria prevention measures include keeping doors and windows closed in the evening, emptying/cover water sources.

**FIGURE 2:**
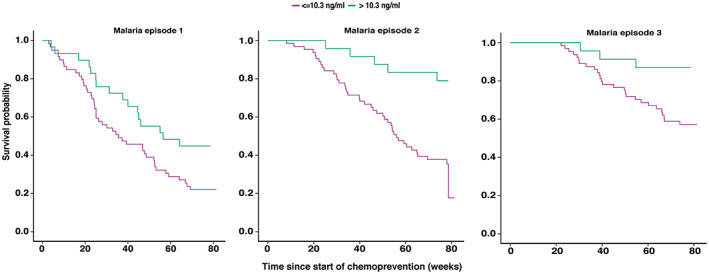
Kaplan‐Meier curves for HIV‐unexposed participants who received dihydroartemisinin‐piperaquine and had piperaquine concentrations measured (*n* = 89), stratified by piperaquine concentration. Time indicates time from beginning of chemoprevention to the subsequent malaria episode. The green line indicates a concentration greater than >10.3 ng/ml piperaquine preceding the malaria episode and purple line indicate a preceding PQ concentration of less than or equal to 10.3 ng/ml.

There were 500 PQ concentrations measured at the time of malaria (*n* = 283, 56.6%) or at monthly routine visits (*n* = 217, 43.4%) from 89 HIV‐unexposed children who received DP. The median PQ concentration was 5.6 ng/ml (range: LLOQ – 316 ng/ml). Children with a preceding PQ concentration of greater than 10.3 ng/ml had a lower cumulative hazard of repeated malaria events compared to when the preceding PQ concentration was less than or equal to 10.3 ng/ml (Figure 2).

Height, weight, and age measurements were available from 27,890 (94.8%) of monthly study visits. Over 88% of measurements were below the WHO defined mean ZHA and 50% met the criteria for stunting (ZHA < ‐2) at least once. Children who never developed malaria between 6 months and 3 years of age trended towards being taller than those who had malaria greater than or equal to one time (92.7 cm vs. 91.6 cm, *p* = 0.35; Figure S2). Non‐stunted children at the start of chemoprevention who became stunted by 3 years of age had a trend toward a higher average number of malaria episodes compared with children who remained non‐stunted (median [5th‐95th percentiles] = 14 [3–29] vs. 12 [1–26], *p* = 0.18; Figure S3).

### Parametric survival analysis

**FIGURE 3:**
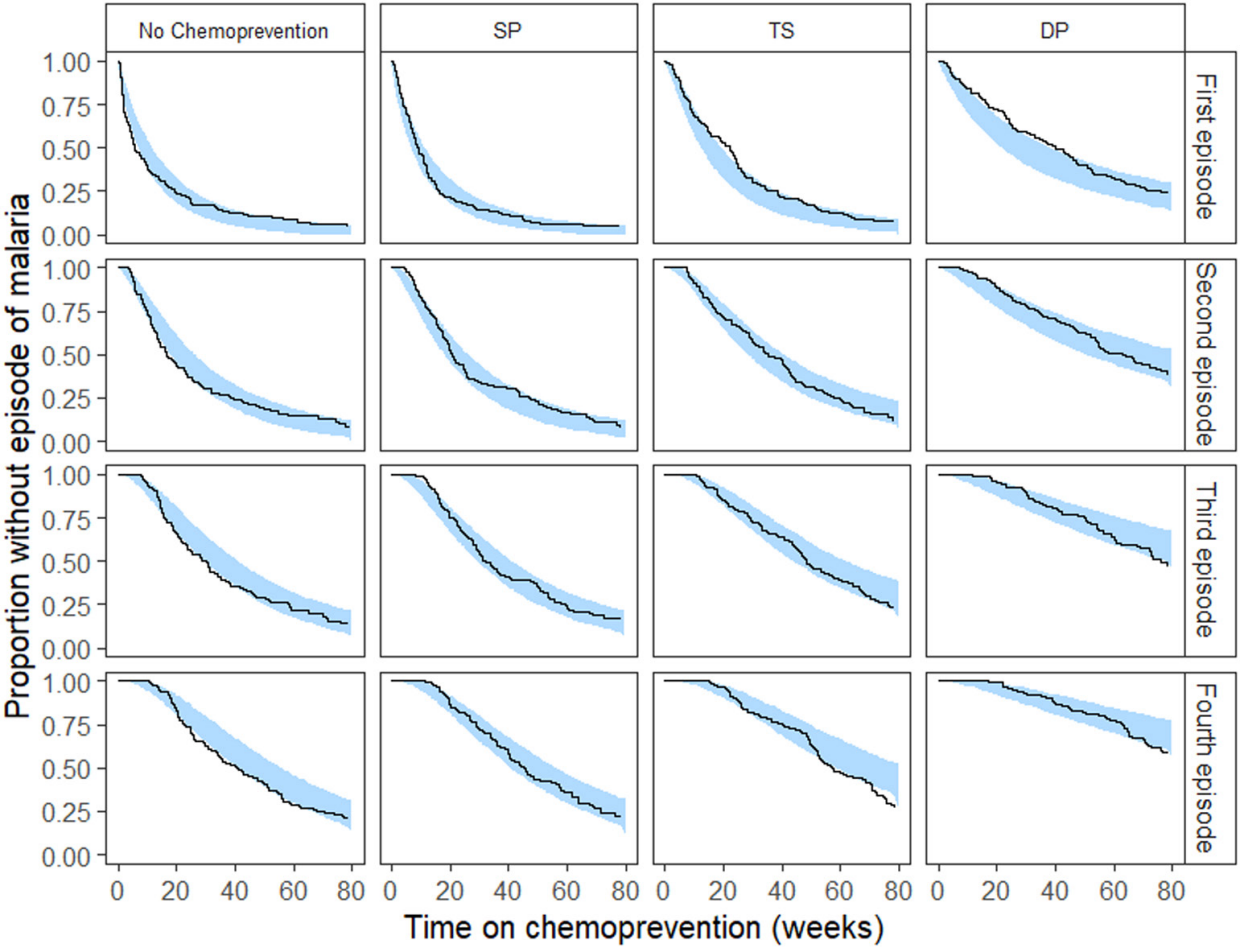
Kaplan‐Meier visual predictive check of the parametric survival model for the first four malaria episodes. The observed data is denoted by the black and the 95% confidence interval of the simulated data is the blue shaded area. Each row of graphs describes survival for a consecutive malaria episode (labels on right side). Each column corresponds to a chemoprevention regimen. DP, dihydroartemisinin‐piperaquine; SP, sulfadoxine‐pyrimethamine; TS, trimethoprim‐sulfamethoxazole.

An exponential survival distribution best predicted malaria for the no chemoprevention and SP regimens (Figure 3). A Weibull distribution provided the best survival distribution for TS (ΔOFV = −173) and DP (ΔOFV = −97.6), compared to an exponential distribution. After controlling for the prevention arm, socioeconomic tertile, and HIV exposure, greater ZHA (ΔOFV = −9.3) was associated with a reduced hazard of malaria (Table 2). ZHA and WAZ were correlated (correlation = 0.67, *p* value < 0.001) and provided similar statistical significance and effect size. However, uncertainty in the covariate parameter estimate was higher (% relative standard error [%RSE] = 77.4) when WAZ was included compared to ZHA (%RSE = 17.8) and ZHA was included in the final model. Age, WHZ, weight, and sex were not associated with malaria hazard. The final survival function is shown in Equation 2.

(2) $$\begin{array}{c} h\left(t\right)=h_{0}\left(t\right)\times \left(1+\theta_{1}\times (\mathrm{ZHA}-\left(-1.32\right)\right)\times \left(1+\theta_{2}\times \mathrm{HIV}\;\text{exposed}\right)\times \left(1+\theta_{3}\times \text{Highincome}\right)\times e^{\eta } \end{array}$$

where *h*(*t*) is the hazard; *h*_0_(*t*) is the baseline hazard, −1.32 is the median ZHA of the population, and high income refers to the socioeconomic tertile with the highest income with low and middle income as the reference category, and *η* is the interindividual variability term.

**TABLE 2:** Parameter estimates for parametric survival models predicting malaria

Parameter	Value (95% CI)^a^	Between subject variability (95% CI)^a^
Parametric survival analysis		
No chemoprevention and SP: Hazard (1/week)	0.102 (0.093, 0.112)	55. 6% (46.2, 64.5)
TS: Hazard (1/week)	0.067 (0.057, 0.077)	78.6% (65.0, 94.7)
TS: Shape parameter of Weibull	1.55 (1.44, 1.66)	
DP: Hazard (1/week)	0.033 (0.025, 0.042)	137% (105, 182)
DP: Shape parameter of Weibull	1.39 (1.26, 1.51)	16.1% (4.12, 22.0)
Covariate effects on hazard		
Height‐for‐age z‐score	−0.075 (−0.121, −0.0314)	
HIV‐exposed^b^	−0.236 (−0.348, −0.104)	
Socioeconomic status		
Low + middle income	Ref	
High income	−0.336 (−0.435, −0.227)	
Pharmacokinetic/pharmacodynamic analysis for piperaquine		
Hazard (1/week)	0.0494 (0.031, 0.080)	78.9% (13.8, 145)
Shape parameter of Weibull	1.30 (1.01, 1.58)	
Covariate effect on hazard^c^		
PQ concentration > 10.3 ng/ml	−0.358 (−0.656, −0.228)	
Height‐for‐age z‐score	−0.355 (−0.482, −0.189)	

Abbreviations: CI, confidence interval; DP, dihydroartemisinin‐piperaquine; PQ, piperaquine; RSE, relative standard error; SP, sulfadoxine‐pyrimethamine; TS, trimethoprim‐sulfamethoxazole.

^a^ The 95% confidence intervals (CIs) were obtained.

^b^ HIV‐exposed indicates children who are HIV‐uninfected but were born to HIV‐infected mothers. Covariate relationship is as follows: $\begin{array}{c} \;h\left(t\right)=h_{0}\left(t\right)\times \left(1+\theta_{1}\times \left(\mathrm{ZHA}-\left(-1.32\right)\right)\right)\times \left(1+\theta_{2}\times \mathrm{HIV}\;\text{exposed}\right)\times \left(1+\theta_{3}\times \text{High income}\right)\times e^{\eta } \end{array}$

^c^ Covariate relationship is given below (if PQ >10.3, PQ concentration > 10.3 equals 1, otherwise PQ concentration > 10.3 is 0): $\begin{array}{c} h\left(t\right)=h_{0}\left(t\right)\times \left(1+\theta_{1}\times \mathrm{PQ}\;\text{Concentration}>10.3\right)\times e^{\theta_{2}\left(\mathrm{HAZ}+1.1\right)}\times e^{\eta }. \end{array}$

Compared to no chemoprevention, there was no difference in hazard of malaria in the SP arm, and these study arms were combined in the analysis. For the TS arm, although there was a 77% reduction in the hazard (HR [95% CI] 0.23 [0.18–0.31]) of malaria within a month of starting chemoprevention or developing an episode of malaria, compared to no chemoprevention or SP, this benefit decreased with time and the hazard of malaria for children who received TS was similar to those who received no chemoprevention or SP after 3.5 months of drug. Compared to no chemoprevention or SP, children who received DP had a lower risk of malaria throughout chemoprevention, but the benefit decreased over time, with a 35% lower hazard of malaria after 18 months of chemoprevention (HR [95% CI] 0.65 [0.41–0.97]; Figure S7). Independent of the chemoprevention arm, HIV‐exposed children had a 21% lower hazard of malaria compared with HIV‐unexposed children (HR 0.79, 95% CI 0.66–0.91; Figure S1). Each standard deviation decrease in the ZHA was associated with an 8.0% (HR 1.08, 95% CI 1.04–1.12) increase in malaria hazard.

### Subanalysis using PQ concentration data

Among individuals with PQ concentration data, a PQ concentration greater than 10.3 ng/ml was associated with a 36% (HR 0.64, 95% CI 0.23–0.99) decreased hazard of subsequent malaria (ΔOFV −5.6) compared to children whose most recent PQ concentration was less than or equal to 10.3 ng/ml. After accounting for PQ concentrations greater than 10.3 ng/ml, a ZHA less than −2 was associated with a twofold increased malaria hazard (HR: 2.1, 95% CI 1.6–3.0) compared to a ZHA of zero (Table 4). All other covariates tested including PQ concentration as a continuous variable were not statistically significantly associated with malaria hazard in the subanalysis (further details in the Supplemental Results).

### Malaria, growth, and nutritional status

All height, weight, and age measurements obtained after the start of chemoprevention were included in height and weight growth models. All significant covariate relationships were identified on the growth rate (KTR) of the Brody function. Cumulative prior malaria episodes (defined as the number of incident malaria episodes which occurred prior to a height measurement), ZHA and WHZ at the start of chemoprevention, and HIV exposure were associated with height (equation listed in the Supplemental Methods; Figure S5). Each additional episode of incident malaria was associated with a 0.4% (95% CI: 0.3% to 0.5%) decrease in growth rate for height (ΔOFV −97). Children with lower ZHA and WHZ at the start of chemoprevention had slower growth throughout the study period (Table 3). Duration of breastfeeding was highly correlated with HIV exposure. As a result, only HIV exposure status was included in the final model. HIV‐exposed children had a 58% lower growth rate compared with HIV‐unexposed children. Chemoprevention arm and socioeconomic status were not associated with the growth rate after accounting for cumulative malaria episodes. Malaria was not significantly associated with growth for weight.

**TABLE 3:** Parameter estimates of the growth model of height

Parameter	Value (95% CI)	Between subject variability (95% CI)
Baseline height (cm)	64.1 (63.2, 64.5)	4.7% (3.61, 4.90)
Length asymptote (cm)	105 (102, 107)	
*α*	0.003 (0.003, 0.004)	23.1% (18.5, 25.9)
*β*	1.67 (1.58, 1.75)	
Proportional error	0.016 (0.015, 0.045)	
Covariate effects		
Cumulative number of malaria episodes on *α*	−0.004 (−0.006, −0.0001)	
HIV‐exposed^a^ on *α*	−0.580 (−0.680, −0.248)	
HIV‐exposed^a^ on *β*	0.131 (0.039, 0.176)	
Baseline height‐for‐age z‐score on *α*	0.057 (0.044, 0.086)	
Baseline weight‐for‐height z‐score on *α*	0.055 (0.033, 0.078)	
Female on baseline height	−0.022 (−0.030, −0.016)	

Abbreviations: CI, confidence interval; RSE, relative standard error.

^a^ HIV‐exposed, indicates children who are HIV‐uninfected but were born to HIV‐infected mothers; growth rate was estimated as = *α* × Age^*β*^. The covariate relationship is given below: $\begin{array}{c} \text{Growth rate}=\alpha \times \left(1+\theta_{1}\times \text{Cumulative}\_\text{malaria}\right)\times \left(1+\theta_{2}\times \text{HIVexposed}\right)\\ \;\times \left(e^{\theta_{3}\times \left(\text{Baseline}\;\mathrm{ZHA}-\left(-1.15\right)\right)+\theta_{4}\times \left(\text{Baseline}\;\mathrm{WHZ}-\left(-0.3\right)\right)}\right)\times {\mathrm{Age}}^{\beta \times \left(1+\theta_{5}\times \text{Female}\right)\times \left(1+\theta_{6}\times \text{HIVexposed}\right)}\times e^{\eta }. \end{array}$

### Simulations

Simulations of the final survival models revealed that by 24 months of age, stunted children without chemoprevention were predicted to experience one additional malaria episode compared with children with a ZHA of zero (Table 4). DP was predicted to avert the greatest number of malaria episodes compared with no chemoprevention or SP, but the magnitude of these differences was dependent on PQ levels and nutritional status (Table 4). A preceding PQ concentration greater than 10.3 ng/ml was the most protective, with a 66% reduction in the median number of malaria episodes by 24 months of age for both stunted and non‐stunted children as compared with PQ concentrations less than or equal to 10.3 ng/ml.

**TABLE 4:** Number of malaria episodes for stunted and non‐stunted children by piperaquine concentration

Age (months)					DP			
	No chemoprevention		TS		≤10.3 ng/ml		>10.3 ng/ml	
	Stunted	Non‐stunted	Stunted	Non‐stunted	Stunted	Non‐stunted	Stunted	Non‐stunted
6–12	3 (0–6)	2 (0–5)	2 (0–5)	2 (0–5)	2 (0–6)	1 (0–3)	1 (0–4)	0 (0–2)
6–18	5 (1–10)	4 (1–9)	4 (1–10)	3 (0–9)	4 (1–10)	2 (0–6)	2 (0–7)	1 (0–4)
6–24	7 (2–14)	6 (2–12)	6 (1–14)	5 (1–14)	6 (1–15)	3 (0–8)	4 (0–11)	2 (0–6)

*Note*: Numbers are presented as mean (5th – 95th percentiles of the simulated data); stunted: height‐for‐age z‐score = −2.1 non‐stunted: height‐for‐age Z‐score of 0; Children were assumed to be HIV‐unexposed, in the low socioeconomic category.

Abbreviations: DP, dihydroartemisinin‐piperaquine; TS, trimethoprim‐sulfamethoxazole.

When quantifying the impact of malaria on height and ZHA from 6 months to 3 years of age, we simulated populations with no malaria episodes, five episodes, 11 episodes, and 17 episodes. Compared with children with no malaria episodes there was a 1.3%, 3.2%, or 5.2% increase in the number of stunted children if children developed 5, 11, or 17 malaria episodes, respectively (Figure S4, Table S3).

## DISCUSSION

Using parametric survival and growth modeling, we quantified the interplay between malaria and malnutrition in Ugandan children in a high perennial malaria transmission setting. Our findings indicate that improved malaria control in high transmission settings could improve overall health by reducing malnutrition, but that even when a child receives active malaria chemoprevention the risk of malaria is increased in malnourished children compared with nourished children. This indicates that further optimization of malaria chemoprevention is needed for malnourished children. In addition, we show that clinical trials and associated pharmacokinetic/pharmacodynamic (PK/PD) studies of drug‐based malaria prevention interventions should prioritize enrolling malnourished children and they should also incorporate outcomes, such as measures of malnutrition to best evaluate the intervention's health impacts.

Malnutrition is highly prevalent in Africa, including in this cohort of Ugandan children. The estimated national prevalence of stunting in Ugandan children less than 5 years of age is 28.5%,^2^ similar to the 25% of children who were stunted at the start of chemoprevention in these studies. Chronic malnutrition, as measured by low ZHA, has been reported as a risk factor for malaria.^5^, ^8^, ^30^, ^31^ However, the use of parametric survival analysis in this large cohort with rich longitudinal data and high malaria incidence allowed us to quantify how decreasing ZHA incrementally increased the risk of malaria. This continuous relationship indicates that even an incremental improvement in nutritional status could reduce a child's malaria risk.

Multiple potential mechanisms for an association between malnutrition and malaria have been proposed, including malnutrition leading to reduced immunity to malaria by lowering anti‐*P. falciparum* IgG antibody response, or lower anti‐malarial drug exposure due to insufficient weight‐based dosing or lower oral bioavailability.^3^, ^10^, ^28^, ^29^, ^32^ Several studies in young children have identified 11–39% lower bioavailability to antimalarials associated with malnutrition.^29^, ^33^, ^34^ It is also possible that if malnourished children have lower *P. falciparum* immunity, they will need even higher drug exposure for full malaria protection.^32^ In Uganda, individuals considered to have lower immunity to *P. falciparum*, including children and primigravida pregnant women, demonstrated lower treatment responses for acute malaria or required higher drug concentrations for full malaria prevention.^32^, ^35^ Although dosing times for DP were not available for precise PK exposure estimates, after incorporating available PQ data, malnutrition remained a risk factor for malaria indicating our current methods to quantify relationships between malnutrition and PK do not fully account for the higher malaria risk for malnourished children. Modification of dose or frequency of DP have been proposed to increase chemoprevention efficacy,^27^, ^36^, ^37^ and optimization of chemoprevention regimens to account for nutritional status may be needed to counteract the increased risk of malaria in these children.

Complementary to this finding, the Weibull distribution (increasing hazard of malaria over time) best predicted malaria hazard in the TS and DP arms, whereas in the no chemoprevention and SP arms, the hazard was constant. These differences in malaria risk trajectory by the chemoprevention arm may have been due to lower adherence to the study drugs as the study progressed or insufficient weight‐based doses as the child aged resulting in suboptimal drug exposure. PQ concentrations decreased over time as efficacy decreased in the DP arm. Dose optimization to compensate for developmental changes in drug metabolism as well as adherence interventions could be needed to improve malaria chemoprevention.

Other studies have suggested malnutrition could be a confounder for socioeconomic factors that could increase malaria risk (e.g., housing construction, parental access to LLINs, or education).^12^, ^38^, ^39^ In our analysis, ZHA remained a significant predictor of malaria hazard after controlling for chemoprevention regimen and socioeconomic status, indicating that malnutrition is likely affecting malaria risk through a different mechanism.

Although HIV‐exposed and ‐unexposed children received their care at the same study clinic and were enrolled over a similar period of time, HIV‐exposed children had a 21% lower risk of malaria compared to HIV‐unexposed children throughout the study period. A biological cause for decreased risk of malaria associated with HIV exposure is unlikely. Prior studies have found no difference in the burden of malaria between HIV‐exposed and HIV‐unexposed infants.^40^ However, HIV‐exposed children may have been more adherent to study drugs, had shorter follow‐up durations including fewer days in the relatively higher transmission season as compared to HIV‐unexposed children (175 vs. 253 days),^41^ may have been more likely to live in urban areas with lower malaria burden or reside in homes with lower parasite burdens as family members with HIV may also have taken TS compared with HIV‐unexposed participants. We controlled for differences in malaria hazard by HIV exposure, but do not expect stratifying malaria chemoprevention interventions by HIV exposure status will be needed.

We found each additional malaria episode was associated with a decreased subsequent growth rate for height by 0.4%. As malaria episodes increased, so did the risk of stunting by 3 years of age. Consistent with this finding, effective malaria prevention with LLIN has been associated with significant weight and length gains in children.^15^ The mechanism of the relationship between malaria and growth rate is unclear, and further investigation is needed to understand the causal pathway. It is possible that without addressing concurrent illnesses, such as malaria,^42^, ^43^ nutritional interventions targeting malnutrition may not have maximum impact. Full use of longitudinal growth data to predict the impact of malaria prevention on healthy growth and development will be a key tool for quantifying the benefits of malaria control in endemic regions.

This study has some limitations. First, we could not independently assess the role of breastfeeding on growth, due to confounding with HIV‐exposure. As measured, breast‐feeding did not improve growth predictions in our models, although this has been an important predictor in prior studies which assessed growth in sub‐Saharan African infants.^44^, ^45^ Second, accurate information on dosing times and adherence to study drugs was not available. As a result, we could not accurately quantify the PKs of PQ in the study population or develop a population PK/PD model, which would allow us to safely recommend optimized DP regimens for malaria chemoprevention in children less than 2 years of age. Further research in this area is needed. We note the parametric survival model slightly overpredicted the hazard of incident malaria during the initial study drug period for DP while accurately predicting the overall malaria hazard. We hypothesize this finding was due to increased adherence to DP at the beginning of the study, but further studies will be needed to evaluate these PK/PD relationships. Finally, given limitations in the available data, we could not identify the pathophysiologic mediators of the relationships between malaria and malnutrition, and this is an area which warrants future research.

In conclusion, this analysis supports a bidirectional relationship between malnutrition, as measured by ZHA, and an increased risk of recurrent malaria. We found that the relationship between increased risk of malaria with lower ZHA persisted despite the choice of chemoprevention regimen, even when the most efficacious malaria chemoprevention regimen (DP) was used. Our findings suggest that optimizing chemoprevention regimens for high‐risk groups like malnourished young children, will be essential to maximize efficacy of any chemoprevention option. Further research is needed to identify the optimal chemoprevention regimens for young children with malnutrition and to evaluate how treating chronic malnutrition during infancy could improve the burden of malaria and overall health outcomes in young children.

## AUTHOR CONTRIBUTIONS

A.M.A., E.W., E.H., G.D., and R.M.S. wrote the manuscript. A.M.A., E.W., E.H., G.D., and R.M.S. designed the research. A.M.A., E.W., and E.H. performed the research. A.M.A., E.W., R.M.S., and E.H. analyzed the data.

## FUNDING INFORMATION

This study was supported by the Bill and Melinda Gates Foundation OPP1191117, and the National Institutes of Health KL2 TR001870‐04.

## CONFLICT OF INTEREST

The authors declared no competing interests for this work.

## Supporting information


Appendix S1

Appendix S2

Appendix S3

Appendix S4
