# Supplementary material for: Interplay among malnutrition, chemoprevention, and the risk of malaria in young Ugandan children: Longitudinal pharmacodynamic and growth analysis
Source: CPT Pharmacometrics Syst Pharmacol. 2023 Mar 14;12(5):656–67. doi: 10.1002/psp4.12892 (PMC10196432; doi:10.1002/psp4.12892)
Supplement: Supplementary file 1 — Appendix S1 [file PSP4-12-656-s002.docx]

**Supplementary methods:**

**WHO Anthro z-score calculation:**

WAZ values less than -6 or greater than 5, ZHA values less than -6 or greater than 6 or WHZ values less than -5 or greater than 5 are not validated in the WHO nutrition metrics and these values were excluded from the analysis.^1^

**Pharmacodynamic model – consideration of interval censoring**

Durations of fever and parasitemia prior to malaria diagnosis were unknown. However, we were not able to use interval censoring to predict the earliest time of parasite emergence as currently available semi-mechanistic models used to predict parasite emergence from the liver were based on data from non-malaria immune adults, and have not provided realistic parasite emergence intervals for highly malaria exposed young Ugandan children.^2^ Predicting the time of parasite emergence from adult non-immune data is further complicated by chemoprevention, which may slow parasite replication rates by variable amounts based on adherence and parasite drug resistance. Given the substantial uncertainty, we used the time to the clinical diagnosis of malaria as our outcome as this is the standard definition used for antimalarial treatment efficacy and is the outcome of interest for malaria chemoprevention, including the parent clinical trials.^3–5^

**Sub-Analysis using data from participants with PQ concentrations**

The covariates tested for this model included PQ concentration, a binary covariate of PQ concentration >10.3 ng/mL, use of non-pharmacologic malaria prevention methods, derived socioeconomic tertile, age, ZHA, WAZ and WHZ. Maintaining a PQ concentration >10.3 ng/mL has previously been associated with 95% protection against malaria parasitemia among pregnant Ugandan women from the same region.^6^ As protective PQ concentrations may vary by region due to local parasite drug resistance patterns, a protective PQ cut off from the same geographic region was selected as the target. However, we explored higher concentrations associated with protection from malaria for seasonal malaria chemoprevention in Burkina Faso (13-17 ng/mL) and from Uganda in a region which received indoor residual spraying of insecticide (15.4 ng/mL).^2,7^

**Growth Models**

Brody growth model:

$Height or weight=LAINF-\left( LAINF-S0 \right) \mathcal{e}^{\left( -\left( KTR \right) \right)}$ (Eq 1)

Where LAINF is the maximum height or weight at 3 years of age, S0 is the height or weight at 6 months of age, and KTR is the growth rate as a function of age (Eq 2).

$KTR= \alpha{Age}^{\beta} \mathcal{e}^{\eta}$ (Eq 2)

Where $\alpha$is the typical growth rate, $\beta$is the parameter for the age function, and η is the random interindividual variability. Random interindividual variability was also included for LAINF and S0. Covariates were investigated on LAINF, S0, and the $\alpha$ and β parameters of KTR using Stepwise Covariate Model Building (SCM).

**Supplementary Results:**

**Malaria, Growth and Nutritional Status**

Cumulative prior malaria episodes (defined as the number of incident malaria episodes which occurred prior to a height measurement), ZHA and WHZ at the start of chemoprevention, and HIV exposure were associated with height as described in equation 3.

$KTR= \alpha\times\left( 1+ \theta_{1}\times Cumulative malaria \right)\times\left( 1+ \theta_{2}\times HIV exposed \right) \times\left( \mathcal{e}^{\theta_{3}\times(Baseline ZHA1-(-1.15)+\theta_{4}\times(Baseline WHZ-0.3)} \right)\times{Age}^{\beta\times(1+ \theta_{5}\times Female)\times\left( 1+ \theta_{6}\times HIVexposed \right)}\times\mathcal{e}^{\eta}$ (Eq. 3)

Where: $\alpha$is the baseline growth rate, $\theta_{1}$ is the parameter for time varying cumulative malaria, $\theta_{2}$ is the parameter for HIV-exposed status, $\theta_{3}$ and $\theta_{4}$ are parameters for ZHA and WHZ at the start of chemoprevention, $\beta$ is the baseline parameter for age on the growth rate, $\theta_{5}$ is the parameter for female sex, and $\theta_{6}$ is the parameter for HIV exposure on the age varying growth rate.

**Sub-analysis using PQ concentration data**

In a sensitivity analysis we incorporated high income wealth tertile, as this was a significant covariate in the parametric survival model using data from all study participants. In this larger model, the PQ concentration and ZHA covariate effects and Weibull shape parameter changed by <10% however the baseline hazard diverged more greatly from the estimate in the model with the full trial data. As a result, wealth tertile was not included a priori in the PQ concentration sub analysis. Finally, increasing the PQ concentration cut off to other literature values including 15.4 ng/mL or 17 ng/mL did not improve model fit and increased between subject variability and parameter uncertainty, so a PQ cut-off of 10.3 ng/mL was retained in the final model. ^2,7^

**Supplementary tables and figures**

| **Table S1. Characteristics of study participants at start of study drug by HIV-exposure status.** | | | | | |  |
| --- | --- | --- | --- | --- | --- | --- |
| Characteristic |  | HIV-exposure* | | | |  |
|  |  | Unexposed | | Exposed | | |
| Age in months, median (2.5^th^, 97.5^th^ percentile) |  | 6.0 (6.0, 6.3) | | 10.0 (6.5, 18.0) | | |
| Weight in kg, median (2.5^th^, 97.5^th^ percentile) |  | 7.5 (5.7, 9.5) | | 8 (5.3, 10.1) | | |
| Weight-for-age z-score, median (2.5^th^, 97.5^th^ percentile) |  | -0.2 (-2.8, 1.6) | | -1.2 (-3.8, 0.9) | | |
| Height-for-age z-score, median (2.5^th^, 97.5^th^ percentile) |  | -0.8 (-3.4, 1.4) | | -1.7 (-4.6, 0.6) | | |
| Weight-for-height z-score, median (2.5^th^, 97.5^th^ percentile) | | 0.4 (-1.7, 2.4) | -0.4 (-3.0, 1.7) | |  |  |
| Underweight^a^, n (%) |  | 27 (6.9) | | 46 (24.7) | | |
| Stunted^a^, n (%) |  | 70 (17.8) | | 74 (39.8) | | |
| Wasted^a^, n (%) |  | 5 (1.3) | | 13 (7.0) | | |
| Income strata, n (%) |  |  | |  | | |
| Low |  | 156 (39.7) | | 59 (31.7) | | |
| Middle |  | 143 (36.4) | | 51 (27.4) | | |
| High |  | 94 (23.9) | | 76 (40.9) | | |

*HIV-exposed indicates children who are HIV-uninfected but were born to HIV-infected mothers; ^a^Underweight: weight-for-age z-score < -2; Stunted: height-for-age z-score < -2; Wasted: weight-for-height z-score < -2.

| **Table S2. Number of children who had respective number of malaria episodes by treatment arm.** | | | | | |
| --- | --- | --- | --- | --- | --- |
| **Malaria episodes** | **No chemoprevention** | **Daily TS** | **Monthly SP** | **Monthly DP** | **ALL** |
| 0 | 10 | 20 | 11 | 45 | 86 |
| 1 | 9 | 16 | 13 | 25 | 63 |
| 2 | 12 | 17 | 13 | 15 | 57 |
| 3 | 10 | 10 | 13 | 14 | 47 |
| 4 | 10 | 19 | 10 | 8 | 47 |
| 5 | 10 | 10 | 14 | 5 | 39 |
| 6 | 13 | 10 | 8 | 11 | 42 |
| 7 | 11 | 12 | 13 | 4 | 40 |
| 8 | 10 | 5 | 8 | 4 | 27 |
| 9 | 8 | 5 | 6 | 1 | 20 |
| 10 | 9 | 4 | 5 | 3 | 21 |
| 11 | 4 | 3 | 2 | 1 | 10 |
| 12 | 1 | 2 | 7 | 1 | 11 |
| 13 | 8 | 4 | 7 | 6 | 25 |
| 14 | 6 | 2 | 6 | 2 | 16 |
| 15 | 7 | 3 | 2 | 0 | 12 |
| 16 | 3 | 2 | 3 | 0 | 8 |
| 17 | 2 | 1 | 1 | 0 | 4 |
| 18 | 1 | 0 | 2 | 0 | 3 |
| 19 | 0 | 1 | 0 | 0 | 1 |

DP, dihydroartemisinin-piperaquine; SP, sulphadoxine-pyrimethamine; TS, trimethoprim-sulfamethoxazole

| **Table S3. Proportion of stunted children by cumulative malaria episodes at 36 months of age.** | | | | | | | | |
| --- | --- | --- | --- | --- | --- | --- | --- | --- |
|  | No malaria |  | 5 episodes of malaria |  | 11 episodes of malaria |  | 17 episodes of malaria |  |
| Overall | 11.9 (9.5 - 13.8) |  | 13.2 (10.8 - 15.2) |  | 15.1 (12.8 - 16.9) |  | 17.1 (15.0 - 19.3) |  |
| HIV-exposure | | | | | | | | |
| HIV-unexposed | 11.8 (9.2 - 13.8) |  | 13.2 (10.8 - 15.4) |  | 15.1 (13.0 - 16.9) |  | 17.1 (15.0 - 19.2) |  |
| HIV-exposed | 12.0 (10.4 -13.8) |  | 13.2 (11.3 - 14.9) |  | 15.2 (12.8 - 17.2) |  | 17.1 (15.0 - 19.5) |  |

Numbers are presented as mean (5th - 95th percentiles); stunted: height-for-age z-score < -2 non-stunted: height-for-age Z-score ≥ -2. Episodes of malaria: Cumulative number of malaria episodes from start of chemoprevention to 36 months of age. HIV-exposed, indicates children who are HIV-uninfected but were born to HIV-infected mother.

**Table S4.** Weight band-based dosing of the malaria chemoprevention regimens.

| **Weight (kg)** | **Trimethoprim-Sulfamethoxazole**  **daily dosing** | | |
| --- | --- | --- | --- |
|  | 20mg/100mg tabs | 40mg /200mg /5ml susp. | 80mg /400mg tabs |
| < 5 | 1 tab | 2.5 ml | ¼ tab |
| 5-15 | - | 5 ml | ½ tab |
| > 15-30 | - | - | 1 tab |
| **Weight (kg)** | **Dihydroartemisinin-Piperaquine (40mg/320mg tabs)**  **monthly dosing given once a day for 3 consecutive days** | | |
| < 5 | ¼ tab | | |
| 6-10 | ½ tab | | |
| 11-14 | ¾ tab | | |
| 15-19 | 1 tab | | |
| 20-23 | 1 ¼ tab | | |
| 24-25 | 1 ½ tab | | |
| **Weight (kg)** | **Sulfadoxine-Pyrimethamine (500mg/ 25mg tabs)**  **monthly dosing given as a single dose** | | |
| <12 | ½ tab | | |
| 13-18 | ¾ tab | | |
| 19-22 | 1 tab | | |
| 23-25 | 1 ¼ tab | | |


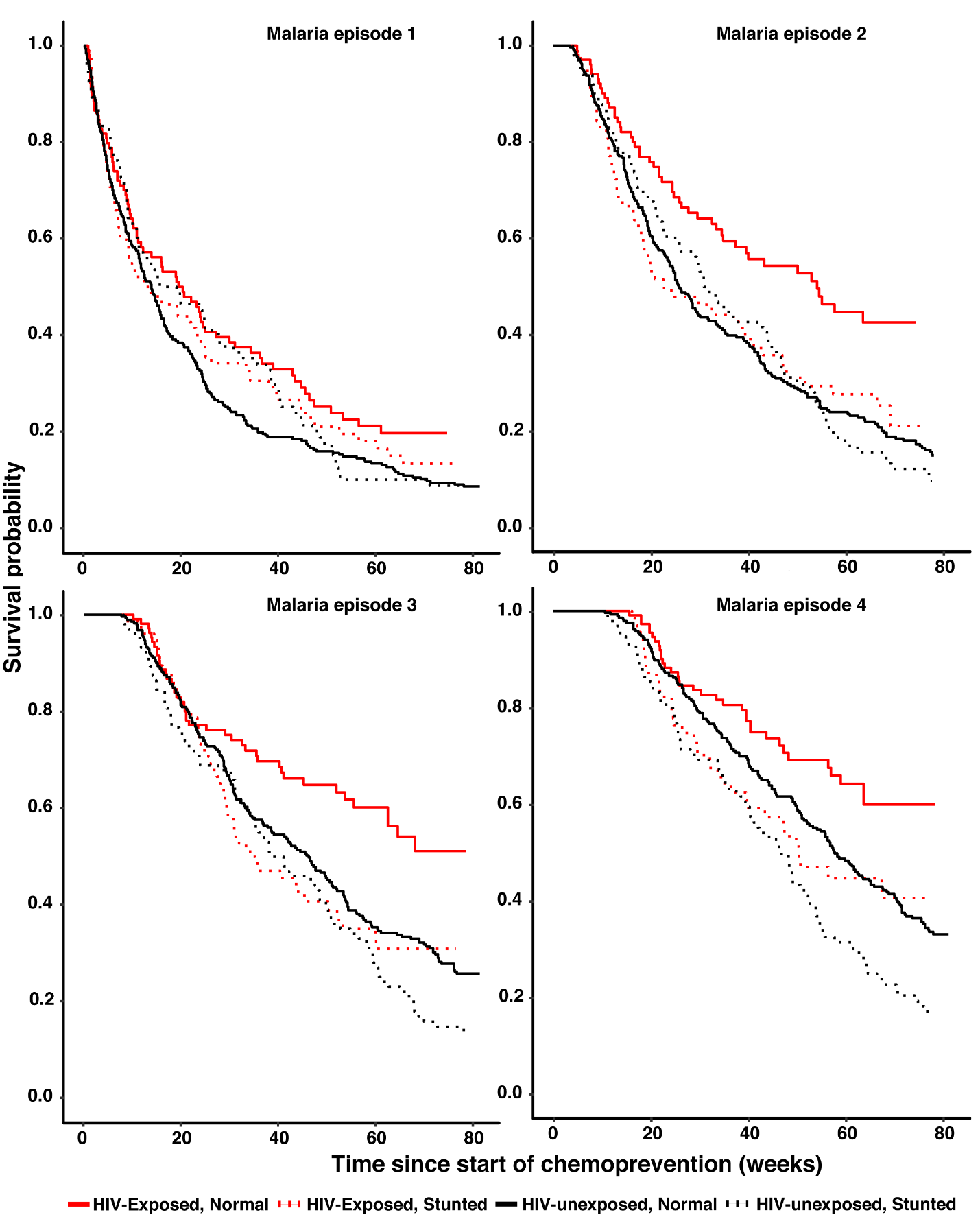


Figure S1. Kaplan Meier curves for the first four malaria episodes stratified by HIV exposure status and nutritional status. Time indicates time from beginning of chemoprevention to the subsequent malaria episode. Solid lines indicate non-stunted children (height-for-age z-score ≥ -2), dashed lines indicate stunted children (height-for-age z-score <-2). HIV-exposed is indicated with red lines and HIV-unexposed is indicated with black lines. HIV-exposed, indicates children who are HIV-uninfected but were born to HIV-infected mothers


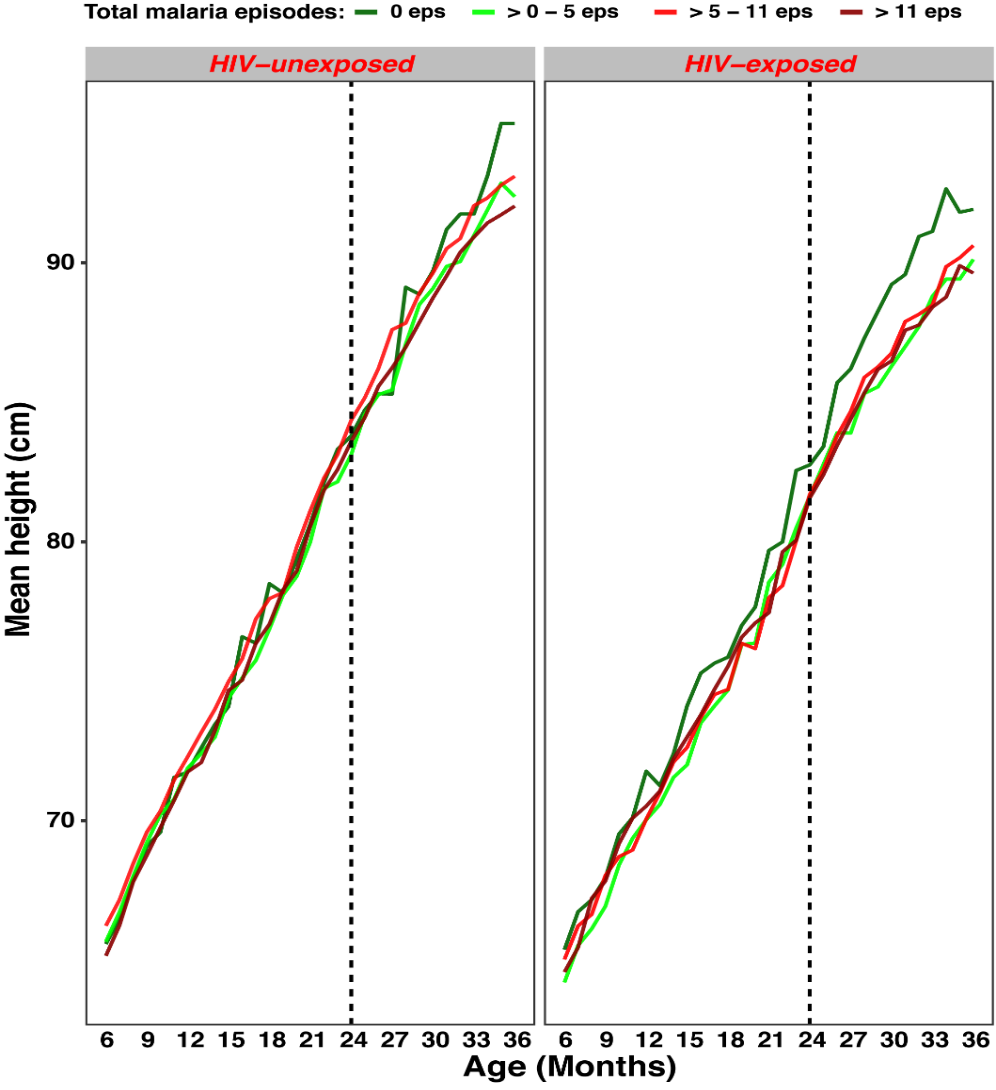


Figure S2. Mean height by total malaria episodes from 6 months to 36 months of age for HIV-unexposed and HIV-exposed children. The dark green line represents children who did not get malaria; the light green line includes children who had 1-5 malaria episodes; the red line represents children who had 6-11 malaria episodes; and the dark red line includes children who had 12 or more malaria episodes. HIV-exposed, indicates children who are HIV-uninfected but were born to HIV-infected mother. The dashed line at 24 months indicates the time at which chemoprevention was stopped.


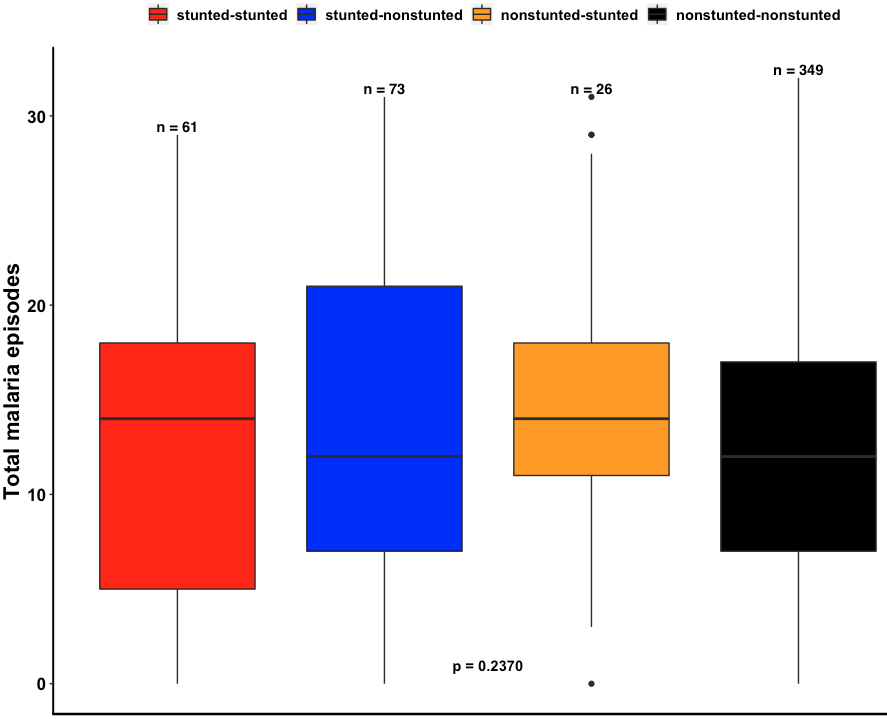


Figure S3. Distribution of total malaria episodes per child from the start of chemoprevention to 36 months of age by nutritional status. The red bar includes children who were stunted at the start of chemoprevention and remained stunted at 36 months of age; the blue bar includes children who were stunted at the start of chemoprevention and were not stunted at 36 months of age; the orange bar includes children who were not stunted at the start of chemoprevention and were stunted by 36 months of age; and the black bar includes children who were not stunted at the start of chemoprevention and were not stunted at 36 months of age. Stunted: height-for-age z-score < -2 non-stunted: height-for-age Z-score ≥ -2.


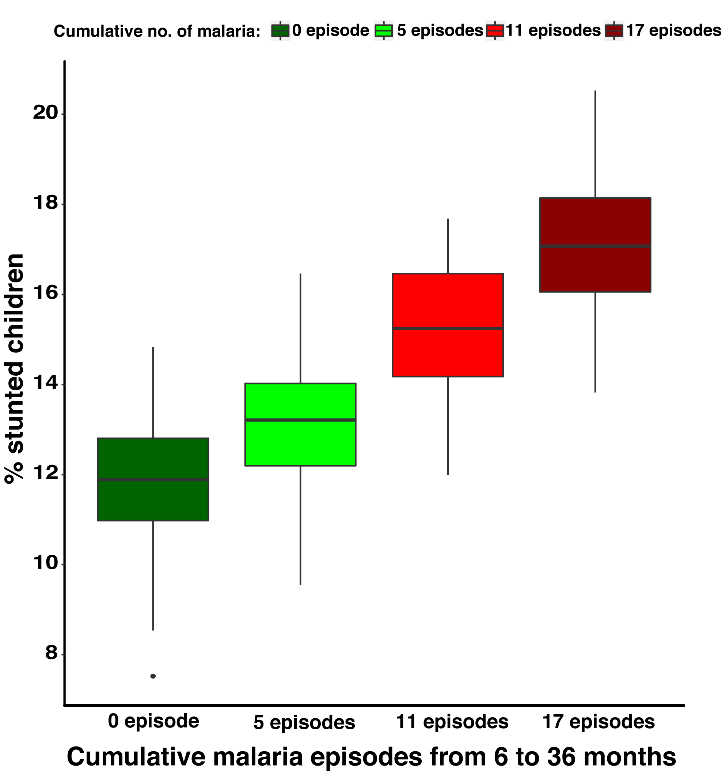


Figure S4. Simulated percentage of stunted children stratified by cumulative number of malaria episodes between 6 and 36 months of age. Stunted is defined as a height-for-age z-score < -2. For these simulations, children were assumed to be HIV-unexposed.


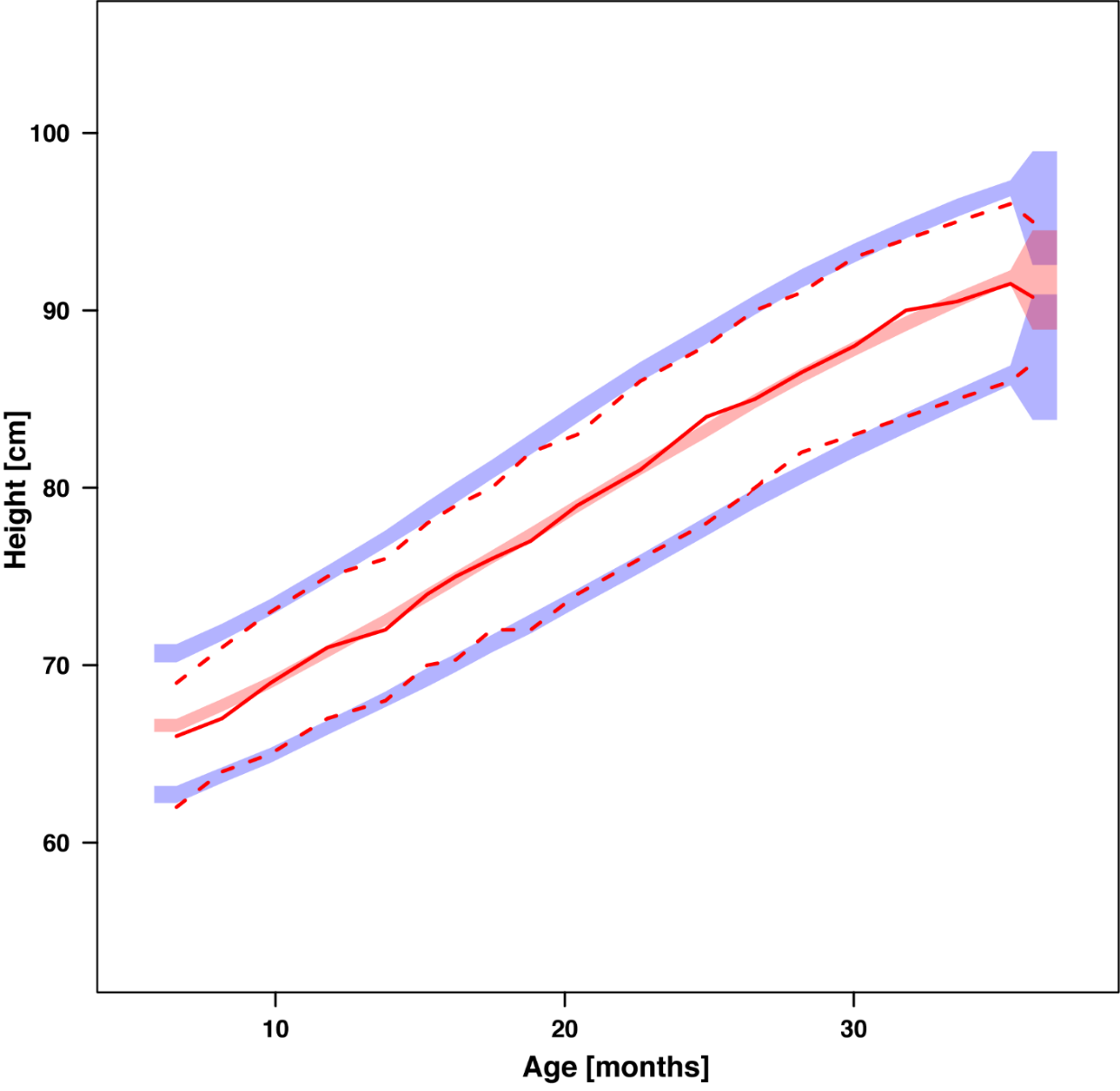


Figure S5. Visual predictive check of the final growth model of height. ﻿The solid and dashed lines are the median and 5th and 95th percentiles of the observed data, respectively. The shaded areas represent the 95% confidence intervals of the model simulated data.


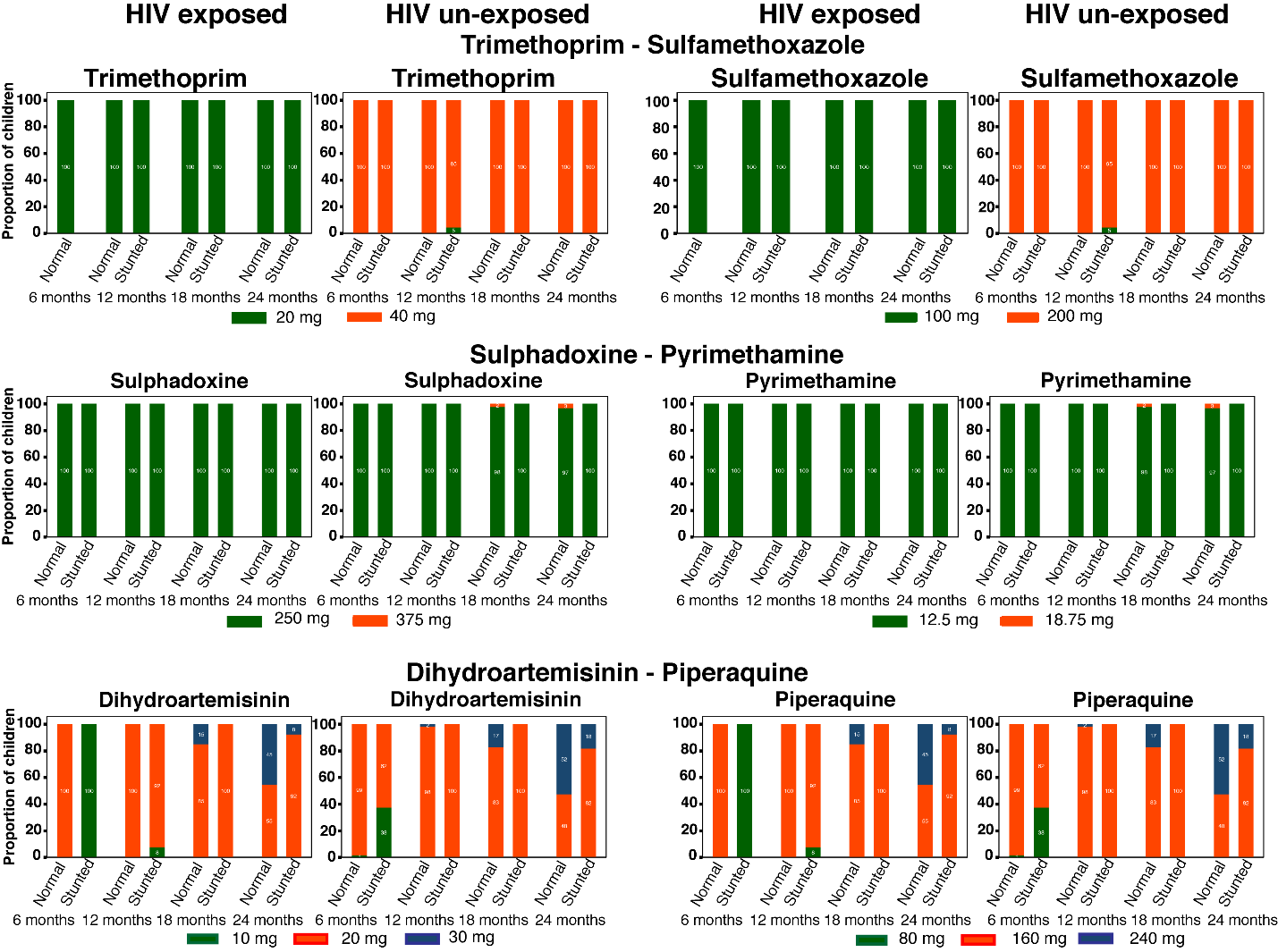


Figures S6: Dosing distribution in children for each prevention combination stratified by nutritional and HIV exposure status.


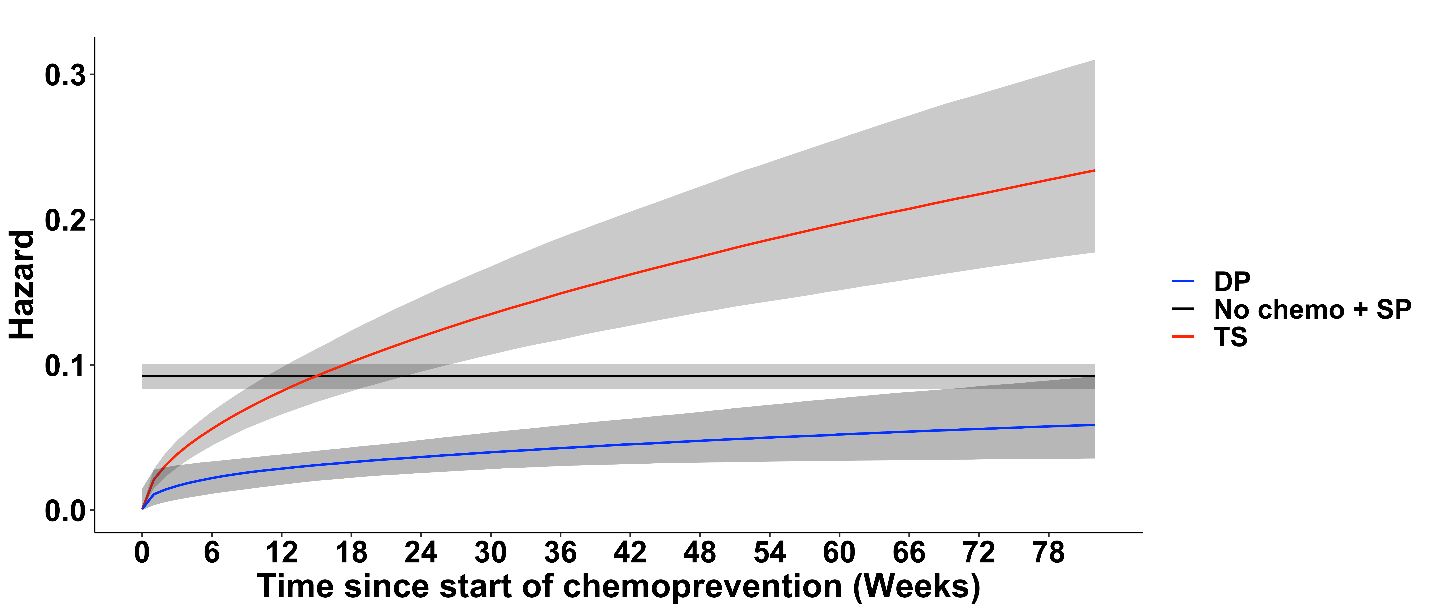


Figure S7. Model derived hazard relationships by treatment arm. The solid lines indicate the median value, and the shaded areas represent the 95% confidence intervals of the model simulated data.

**Supplementary References**

1. World Health Organization *WHO Child Growth Standards - Methods and Development*. (Geneva, Switzerland, 2007).

2. Wallender, E., Ali, A. M., Hughes, E., *et al.* Identifying an optimal dihydroartemisinin-piperaquine dosing regimen for malaria prevention in young Ugandan children. *Nat Commun* **12**, (2021). DOI: 10.1038/s41467-021-27051-8

3. Bigira, V., Kapisi, J., Clark, T. D., *et al.* Protective efficacy and safety of three antimalarial regimens for the prevention of malaria in young Ugandan children: A randomized controlled trial. *PLoS Med* **11**, (2015). DOI: 10.1371/journal.pmed.1001689

4. Kamya, M. R., Kapisi, J., Bigira, V., *et al.* Efficacy and safety of three regimens for the prevention of malaria in young HIV-exposed Ugandan children: A randomized controlled trial. *AIDS* **28**, 2701–2709 (2014). DOI: 10.1097/QAD.0000000000000497

5. World Health Organization *Template Protocol for Therapeutic Efficacy Studies*.

6. Savic, R. M., Jagannathan, P., Kajubi, R., *et al.* Intermittent Preventive Treatment for Malaria in Pregnancy: Optimization of Target Concentrations of Dihydroartemisinin-Piperaquine. *Clin Infect Dis* **67**, 1079–1088 (2018). DOI: 10.1093/cid/ciy218

7. Chotsiri, P., Zongo, I., Milligan, P., *et al.* Optimal dosing of dihydroartemisinin-piperaquine for seasonal malaria chemoprevention in young children. *Nat Commun* **10**, (2019). DOI: 10.1038/s41467-019-08297-9
