# Supplementary material for: Interplay among malnutrition, chemoprevention, and the risk of malaria in young Ugandan children: Longitudinal pharmacodynamic and growth analysis
Source: CPT Pharmacometrics Syst Pharmacol. 2023 Mar 14;12(5):656–67. doi: 10.1002/psp4.12892 (PMC10196432; doi:10.1002/psp4.12892)
Supplement: Supplementary file 2 — Appendix S2 [file PSP4-12-656-s004.docx]

Growth model

$PROBLEM Final model:run2414b

;------------------MODEL & DATA DESCRIPTION------------------------------------------------;

;

$INPUT

$DATA

;

$PRED

;;; S0GENDER-DEFINITION START

IF(GENDER.EQ.1) S0GENDER = 1 ; male

IF(GENDER.EQ.2) S0GENDER = ( 1 + THETA(11))

;;; S0GENDER-DEFINITION END

;;; S0-RELATION START

S0COV=S0GENDER

;;; S0-RELATION END

;;; POWERGROUP-DEFINITION START

IF(GROUP.EQ.0) POWERGROUP = 1 ; HIV negative

IF(GROUP.EQ.1) POWERGROUP = ( 1 + THETA(10))

;;; POWERGROUP-DEFINITION END

;;; POWER-RELATION START

POWERCOV=POWERGROUP

;;; POWER-RELATION END

;;; BASEWHZ_B-DEFINITION START

BASEWHZ_B = EXP(THETA(9)*(WHZ_B - 0.3)) ; Weight-for-age Z score

;;; BASEWHZ_B-DEFINITION END

;;; BASEHAZ_B-DEFINITION START

BASEHAZ_B = EXP(THETA(8)*(HAZ_B + 1.15)) ; Height-for-age Z score

;;; BASEHAZ_B-DEFINITION END

;;; BASEGROUP-DEFINITION START

IF(GROUP.EQ.0) BASEGROUP = 1 ; HIV negative

IF(GROUP.EQ.1) BASEGROUP = ( 1 + THETA(7))

;;; BASEGROUP-DEFINITION END

;;; BASECUMMAL-DEFINITION START

BASECUMMAL = ( 1 + THETA(6)*(CUMMAL)) ; Cumulative malaria

;;; BASECUMMAL-DEFINITION END

;;; BASE-RELATION START

BASECOV=BASECUMMAL*BASEGROUP*BASEHAZ_B*BASEWHZ_B

;;; BASE-RELATION END

;

;Auxological Anthropometric dynamics (PD)

;

TVBASE = THETA(1) ; Base of the power function

TVBASE = BASECOV*TVBASE

BASE = TVBASE*DEXP(ETA(4))

TVPOWER = THETA(4)

TVPOWER = POWERCOV*TVPOWER ; Power of the power function

POWER = TVPOWER*DEXP(ETA(5))

TVHtr = BASE*(TIME**POWER)

Htr = TVHtr*DEXP(ETA(1)) ; Height growth rate coefficient

TVS0=THETA(2)

TVS0 = S0COV*TVS0

S0=TVS0*DEXP(ETA(2))

TVLAINF=THETA(5)

LAINF=TVLAINF*DEXP(ETA(3)) ; Length asymptote

;

;OUTPUT

;

IPRED = LAINF-(LAINF-S0)*DEXP(-(Htr*TIME))

;

W=THETA(3)

IRES=DV-IPRED

IWRES=IRES/W

Y=IPRED*(1.D0+ERR(1)*W)

;

$THETA (0.001,0.00327569) ; 1 [TVBase]

(0,64.1053,100) ; 2 [TVS0]

(0,0.015567) ; 3 [Prop error]

(0.1,0.668383) ; 4 [TVPower]

(90,104.616) ; 5 [TVLAINF]

;

$THETA (-0.036,-0.00388448,0.250) ; 6 Cumulative_Malaria on Base

$THETA (-1,-0.580254,5) ; 7 HIV_status on Base

$THETA (-0.961413399997514,0.0569552,0.961413399997514) ; 8 HAZ on Base

$THETA (-1.20554193350474,0.0553852,1.20554193350474) ; 9 WHZ on Base

$THETA (-1,0.327341,5) ; 10 HIV_status on exponent (power)

$THETA (-1,-0.0223362,5) ; 11 Gender on intercept

;

$OMEGA 0 FIX ; 1 [Htr]

$OMEGA 0.00215365 ; 2 [S0]

$OMEGA 0 FIX ; 3 [LAINF]

$OMEGA 0.0522252 ; 4 [Base]

$OMEGA 0 FIX ; 5 [Power]

;

$SIGMA 1 FIX

;

$ESTIMATION INTER MAX=9990 PRINT=5 METHOD=COND POSTHOC NOABORT

$COVARIANCE MATRIX=R UNCONDITIONAL
