# Supplementary material for: Interplay among malnutrition, chemoprevention, and the risk of malaria in young Ugandan children: Longitudinal pharmacodynamic and growth analysis
Source: CPT Pharmacometrics Syst Pharmacol. 2023 Mar 14;12(5):656–67. doi: 10.1002/psp4.12892 (PMC10196432; doi:10.1002/psp4.12892)
Supplement: Supplementary file 3 — Appendix S3 [file PSP4-12-656-s003.docx]

PKPD

;Sim_start : add to simulation model

;$SIZES NO=500 LIM6=500

;Sim_end

$PROBLEM Final model: run973

;

$INPUT

$DATA

;Sim_start : add to simulation model

IGNORE=(FLGSIM.EQ.1)

;Sim_end

$SUBROUTINE ADVAN=6 TOL=9

$MODEL COMP=(HAZARD)

$PK

;;; BASEPKCUT4-DEFINITION START

IF(CUT5.EQ.0.0000E+00) BASEPKCUT5 = 1 ; Piperaquine concentration <= 10.3 ng/ml

IF(CUT5.EQ.1.0000E+00) BASEPKCUT5 = ( 1 + THETA(3))

;;; BASEPKCUT4-DEFINITION END

;;; BASEHAZ-DEFINITION START

BASEHAZ = EXP(THETA(4)*(HAZ + 1.1)) ; Height-for-age Z score

;;; BASEHAZ-DEFINITION END

;;; BASE-RELATION START

BASECOV=BASEPKCUT5*BASEHAZ

;;; BASE-RELATION END

TVBASE = THETA(1) ; Baseline Hazard

;Covariate relationship

TVBASE = BASECOV*TVBASE

;

BASE = TVBASE*EXP(ETA(1))

TVGAMA = THETA(2) ; Shape of the Weibull function

SHP = TVGAMA

IF(NEWIND.NE.2) TP = 0 ; for RTTE. TP is time of previous event.

; T-TP is time since last event.

; For TTE TP is always 0.

$DES

DEL=1E-6 ; to keep from taking 0**power

DADT(1) = BASE*SHP*(BASE*(T-TP)+DEL)**(SHP-1) ;hazard

$ERROR

;----------RTTE Model------------------------------

IF(NEWIND.NE.2) OLDCHZ = 0 ;reset the cumulative hazard

CHZ = A(1)-OLDCHZ ;cumulative hazard

; from previous time point

; in data set

OLDCHZ = A(1) ;rename old cumulative hazard

SUR = EXP(-CHZ) ;survival probability

DELX = 1E-6

HAZNOW = BASE*SHP*(BASE*(TIME-TP)+DELX)**(SHP-1) ; rate of event

; each time pt

; NB: update with each new model

IF(DV.EQ.0) Y = SUR ;censored event (prob of survival)

IF(DV.NE.0) Y = SUR*HAZNOW ;prob density function of event

IF(ICALL.EQ.4) THEN ; for simulation

CALL RANDOM (2,R)

DV = 0

RTTE = 0

IF(TIME.EQ.83) RTTE = 1 ; for the censored observation at 132 Weeks

IF(R.GT.SUR) THEN

DV = 1

RTTE = 1

ENDIF

ENDIF

IF(TYPE.EQ.1) TP = TIME ; reset time of previous event to current event time

$THETA (0,0.0716704) ; 1 Baseline hazard

$THETA (0,1.09811) ; 2 Shape parameter

$THETA (-2,-0.427256,1) ; 3 Concentration on Baseline hazard

$THETA (-2,-0.358419,1) ; 4 HAZ on Baseline hazard

;

$OMEGA 0.151736 ; 1 BSV

;Sim_start : add/remove for simulation

;$SIMULATION (5988566) (39978 UNIFORM) ONLYSIM NOPREDICTION SUB=100

$ESTIMATION MAXEVAL=9990 METHOD=COND LIKE PRINT=1 MSFO=msfb973 SIGL=9

NSIG=3 NOABORT LAPLACIAN

$COVARIANCE PRINT=E

;Sim_end
