# Supplementary material for: Interplay among malnutrition, chemoprevention, and the risk of malaria in young Ugandan children: Longitudinal pharmacodynamic and growth analysis
Source: CPT Pharmacometrics Syst Pharmacol. 2023 Mar 14;12(5):656–67. doi: 10.1002/psp4.12892 (PMC10196432; doi:10.1002/psp4.12892)
Supplement: Supplementary file 4 — Appendix S4 [file PSP4-12-656-s001.docx]

PD model

;Sim_start : add to simulation model

;$SIZES NO=500 LIM6=500

;Sim_end

$PROBLEM Final model

$INPUT

$DATA

;Sim_start : add to simulation model

IGNORE=(FLGSIM.EQ.1)

;Sim_end

$SUBROUTINE ADVAN=6 TOL=6

$MODEL COMP=(HAZARD)

$PK

;;; BASEHAZ-DEFINITION START

BASEHAZ = ( 1 + THETA(6)*(HAZ + 1.32)) ; Height-for-age Z score

;;; BASEHAZ-DEFINITION END

;;; BASEGROUP-DEFINITION START

IF(GROUP.EQ.0.0000E+00) BASEGROUP = 1 ; HIV negative

IF(GROUP.EQ.1.0000E+00) BASEGROUP = ( 1 + THETA(7))

;;; BASEGROUP-DEFINITION END

;;; BASEWEALTH-DEFINITION START

IF(WEALTH.EQ.1.0000E+00.OR.WEALTH.EQ.2.0000E+00) BASEWEALTH = 1 ; Low + Midle income

IF(WEALTH.EQ.3.0000E+00) BASEWEALTH = ( 1 + THETA(8))

;;; BASEWEALTH-DEFINITION END

;;; BASE-RELATION START

BASECOV=BASEHAZ*BASEGROUP*BASEWEALTH

;;; BASE-RELATION END

TVBASE = THETA(1) ; Baseline Hazard for No chemoprevetion and SP

IF(THERAPY.EQ.2) TVBASE = THETA(2) ; Baseline Hazard for TS

IF(THERAPY.EQ.4) TVBASE = THETA(4) ; Baseline Hazard for DP

TVGAMA = THETA(3) ; Shape of the Weibull function for TS

IF(THERAPY.EQ.4) TVGAMA = THETA(5) ; Shape of the Weibull function for DP

;Covariate relationship

TVBASE = BASECOV*TVBASE

;

BASE = TVBASE*EXP(ETA(1))

IF(THERAPY.EQ.2) BASE = TVBASE*EXP(ETA(2))

IF(THERAPY.EQ.4) BASE = TVBASE*EXP(ETA(3))

SHP = TVGAMA*EXP(ETA(4))

IF(NEWIND.NE.2) TP = 0 ; for RTTE. TP is time of previous event.

; T-TP is time since last event.

; For TTE TP is always 0.

$DES

DEL=1E-6 ; to keep from taking 0**power

DADT(1) = BASE ;hazard

IF(THERAPY.EQ.2.OR.THERAPY.EQ.4) DADT(1) = BASE*SHP*(BASE*(T-TP)+DEL)**(SHP-1)

$ERROR

;----------RTTE Model------------------------------

IF(NEWIND.NE.2) OLDCHZ = 0 ;reset the cumulative hazard

CHZ = A(1)-OLDCHZ ;cumulative hazard

; from previous time point

; in data set

OLDCHZ = A(1) ;rename old cumulative hazard

SUR = EXP(-CHZ) ;survival probability

HAZNOW = BASE ; rate of event

DELX = 1E-6

IF(THERAPY.EQ.2.OR.THERAPY.EQ.4) HAZNOW = BASE*SHP*(BASE*(TIME-TP)+DELX)**(SHP-1)

; each time pt

; NB: update with each new model

IF(DV.EQ.0) Y = SUR ;censored event (prob of survival)

IF(DV.NE.0) Y = SUR*HAZNOW ;prob density function of event

IF(ICALL.EQ.4) THEN ; for simulation

CALL RANDOM (2,R)

DV = 0

RTTE = 0

IF(TIME.EQ.83) RTTE = 1 ; for the censored observation at 132 Weeks

IF(R.GT.SUR) THEN

DV = 1

RTTE = 1

ENDIF

ENDIF

IF(TYPE.EQ.1.AND.THERAPY.EQ.2) TP = TIME ; reset time of previous event to current event time

IF(TYPE.EQ.1.AND.THERAPY.EQ.4) TP = TIME ; reset time of previous event to current event time

;

$THETA (0,0.102387) ; 1 Baseline Hazard for No chemo and SP group

$THETA (0.04,0.0664958) ; 2 Baseline Hazard for TS group

$THETA (0,1.54672) ; 3 Shape parameter for TS group

$THETA (0,0.0328745) ; 4 Baseline Hazard for DP group

$THETA (0,1.3858) ; 5 Shape parameter for TS group

$THETA (-0.222,-0.0746573,0.198) ; 6 HAZ on Baseline hazard

$THETA (-1,-0.23609,5) ; 7 HIV status on Baseline hazard

$THETA (-1,-0.33588,5) ; 8 High economic status on Baseline hazard

;

$OMEGA 0.269129 ; 1 BSV_No chemo+SP

$OMEGA 0.480814 ; 2 BSV_TS

$OMEGA 1.06408 ; 3 BSV_DP

$OMEGA 0.025539 ; 4 BSV_SHP

;Sim_start : add/remove for simulation

;$SIMULATION (5988566) (39978 UNIFORM) ONLYSIM NOPREDICTION SUB=100

$ESTIMATION MAXEVAL=9990 METHOD=COND LIKE PRINT=1 MSFO=msfb729b SIGL=9

NSIG=3 NOABORT LAPLACIAN

$COVARIANCE PRINT=E

;Sim_end
